# New species, new records and key to the species of the *Rhagoveliaitatiaiana* group (Hemiptera, Heteroptera, Veliidae) from Brazil

**DOI:** 10.3897/BDJ.11.e105614

**Published:** 2023-06-12

**Authors:** Oséias Martins Magalhães, Carla Fernanda Burguez Floriano, Felipe Ferraz Figueiredo Moreira

**Affiliations:** 1 Fundação Oswaldo Cruz, Instituto Oswaldo Cruz, Laboratório de Entomologia, Rio de Janeiro, Brazil Fundação Oswaldo Cruz, Instituto Oswaldo Cruz, Laboratório de Entomologia Rio de Janeiro Brazil

**Keywords:** aquatic insects, Gerromorpha, riffle bugs, South America, taxonomy

## Abstract

**Background:**

*Rhagovelia* Mayr, 1865 (Hemiptera, Heteroptera, Veliidae), known as riffle bugs, includes more than 400 species and is commonly found in tropical lotic environments, including coastal marine habitats, such as mangroves and estuaries. Due to the elevated number of species, the fauna from the Americas has been divided into several groups, which facilitates taxonomic studies. Amongst them, the *itatiana* group currently includes two species from the Greater Antilles and five from south-eastern and southern Brazil. Despite the many taxonomic studies developed during the past few decades, new species of *Rhagovelia* are still being discovered in several areas of the continent, including the Atlantic Forest of eastern Brazil.

**New information:**

*Rhagoveliabispoi* sp. n. is described, illustrated and compared with similar congeners. The new species belongs to the *itatiaiana* group and can be diagnosed by the uniformly black mesonotum, the presence of a tuft of setae medially on male abdominal sternum VII, the armature of the male hind femur and the distinctive shape of the paramere. In addition, we present new records of *R.trepida* Bacon, 1948 from the States of Paraná and Santa Catarina and a key to the species of the *itatiaiana* group recorded from Brazil.

## Introduction

Veliidae (Hemiptera, Heteroptera, Gerromorpha) is a worldwide distributed group of semi-aquatic bugs that is especially rich in the Neotropics (Polhemus and Polhemus 2008). The most speciose genus in the region is *Rhagovelia* Mayr, 1865, (Veliidae, Rhagoveliinae), containing almost 200 described American species and being commonly found in lotic environments, sometimes in groups of hundreds of individuals (Padilla-Gil and Moreira 2013, Moreira 2015).

American *Rhagovelia* are distributed into 18 species groups, which are further organised into one grade (non-monophyletic) and five complexes (monophyletic) (Polhemus 1997, Moreira et al. 2012, Padilla-Gil and Moreira 2013). Although not a monophyletic lineage, the *abrupta* grade can be diagnosed by the pronotum of the apterous forms longer than the dorsal length of the eye, but shorter than three times the exposed portion of the mesonotum and with the posterior margin convex (Polhemus 1997).

Five species groups are currently recognised within the *abrupta* grade, namely *cali*, *itatiaiana*, *lucida*, *secluda* and *torquata*. Amongst them, the *itatiaiana* group is recognised by the general blackish colouration with distinctively contrasting orange markings on the pronotum and abdominal laterotergites; the forewing with four closed cells, of which the distalmost two extend into the distal half of the wing (Matsuda 1956, fig. 7); the macropterous females with the dorsal abdominal carinae not evident on mediotergites II and III; and the ventral abdominal sutures simple and unmodified (Polhemus 1997).

There are seven known species in the *itatiaiana* group, namely *R.accedens* Drake, 1957, *R.itatiaiana* Drake, 1953, *R.macta* Drake & Carvalho, 1955, *R.trepida* Bacon, 1956, *R.trianguloides* Nieser & Melo, 1997, *R.mira* Drake & Harris, 1938 and *R.vegana* Drake & Maldonado-Capriles, 1956. The last two species are endemic to the Greater Antilles (Cuba and Dominican Republic), while the other five are restricted to south-eastern and southern Brazil (Polhemus 1997, Padilla-Gil and Moreira 2013, Moreira 2023). Additionally, Padilla-Gil 2012 and Padilla-Gil 2019 described the two Colombian species *R.candelilla* Padilla-Gil, 2012 and *R.mallama* Padilla-Gil, 2019, but considering the general distribution of the group and the issues related to species described by the author in question (see Galindo-Malagón et al. (2021), Galindo-Malagón et al. (2022) for details), this assignment and even the validity of such species need to be verified.

Based on material collected in south-eastern and southern Brazil during the past decade, we present here the description of a new species of the *itatiaiana* group, new records from the States of Paraná and Santa Catarina and a key to the species occurring in the country.

## Materials and methods

The material examined is deposited in the following institutions: Coleção Entomológica do Instituto Oswaldo Cruz, Fundação Oswaldo Cruz, Rio de Janeiro, Brazil (CEIOC); Laboratório de Biologia Aquática, Universidade Estadual Paulista Júlio de Mesquita Filho, Assis, Brazil (LABIA); Museu Nacional, Universidade Federal do Rio de Janeiro, Rio de Janeiro, Brazil (MNRJ, entomological collection destroyed in 2018); Museu de Zoologia, Universidade de São Paulo, São Paulo, Brazil (MZUSP); and National Museum of Natural History, Smithsonian Institution, Washington D.C., USA (NMNH). Methods and terminology follow the standards set in the latest revision of the genus (Polhemus 1997) and subsequent species descriptions. All measurements are given in millimetres.

Digital photographs of the specimens deposited in the CEIOC were obtained using a Leica M205 C stereomicroscope coupled with a Leica DFC450 C digital camera, using the software Leica LAS 4.8.0 for capturing and stacking images. Specimens deposited in the NMNH were photographed with a Cannon EOS 5D digital camera and combined into multi-focal images using Visionary Digital Software. Maps were produced using the software Qgis 2.6.1.

## Taxon treatments

### Rhagovelia bispoi sp. n.

CAAFC51C-818B-5DBD-B141-916E801FBA02

090D3712-A5EB-4E20-A8A9-035805C091CE

#### Materials

- **Type status:** Holotype. **Occurrence:** individualCount: 1; sex: apterous male; occurrenceID: 74FA7FE9-7843-5F2B-AB9A-B0CD6C8FB338; **Taxon:** scientificNameID: *Rhagoveliabispoi*; order: Hemiptera; family: Veliidae; **Location:** continent: South America; country: Brazil; stateProvince: São Paulo; municipality: Iporanga; locality: Parque Estadual Intervales, Riacho Roda D’Água; decimalLatitude: -24.2714; decimalLongitude: -48.4222; geodeticDatum: WGS84; **Event:** verbatimEventDate: 14.XII.2014; eventRemarks: P.C. Bispo leg.; **Record Level:** type: PhysicalObject; collectionCode: CEIOC 82833; basisOfRecord: PreservedSpecimen
- **Type status:** Paratype. **Occurrence:** individualCount: 18; sex: 7 apterous males, 11 apterous females; occurrenceID: DEBD6A2D-8E0E-54AF-852E-FDCE4CE6459C; **Taxon:** scientificNameID: *Rhagoveliabispoi*; order: Hemiptera; family: Veliidae; **Location:** continent: South America; country: Brazil; stateProvince: São Paulo; municipality: Iporanga; locality: Parque Estadual Intervales, Riacho Roda D’Água; decimalLatitude: -24.2714; decimalLongitude: -48.4222; geodeticDatum: WGS84; **Event:** verbatimEventDate: 14.XII.2014; eventRemarks: P.C. Bispo leg.; **Record Level:** type: PhysicalObject; collectionCode: CEIOC 82834; basisOfRecord: PreservedSpecimen

#### Description

**Measurements**. See Table 1.

**Apterous male** (Figs 1, 3a, 4b). General colour black. Head with shiny impressed mid-line and a pair of shiny oblique indentations at base. Antenniferous tubercle brown. Proximal portion of antennomere I yellow; rest of antenna brown. Eye dark reddish-brown. Clypeus, buccula and jugum yellow to yellowish-brown. Labium yellowish-brown, dark-brown at apex. Pronotum black, with transverse yellow band adjacent to head; band at mid-line about 1/3 as long as pronotum, extending over propleuron laterally. Meso- and metanota black (Fig. 1a, c). Meso- and metapleura black, with a small brown mark ventrally on mesopleuron and a larger brown mark dorsally on metapleuron. Proepisternum and proacetabulum light-yellow. Pro-, meso- and metasterna black. Meso- and metacetabula yellow. All coxae yellow. Fore and hind trochanters light-yellow; fore trochanter with a small brown mark distally; middle trochanter yellow to brown proximally, dark-brown to black distally. Fore femur with proximal 1/2 yellow, distal 1/2 dark-brown to black. Middle femur dark-brown to black, darker dorsally. Hind femur dark-brown to black; dorsum with a yellowish-brown mark at base and posterior surface; venter with a large mark, yellow proximally, becoming narrower and brownish distally. All tibiae and tarsi brown to dark-brown. Abdominal mediotergites I–VII brownish-black; V–VII with shiny black spot centrally. Abdominal laterotergites black mesally, yellow laterally. Abdominal sterna II–VI mostly black, orange-brown laterally; VII black, with two yellow spots submesally. Abdominal segment VIII brown, lighter ventrally; pygophore and proctiger brown.

Head short, velvety, with a few long setae anteriorly and adjacent to mesal eye margin. Antennae covered by short brown setae; antennomeres I–II also with a few thicker, longer setae. Antennomeres I–III cylindrical; I curved laterally; IV fusiform. Labium wide, reaching base of mesosternum. Jugum and adjacent portion of proepisternum without black denticles. Pro-, meso- and metanota densely covered by short setae, with longer setae laterally. Pronotum longer than dorsal eye length, shorter than three times exposed portion of mesonotum, with posterior margin convex. Pro-, meso- and metapleura with a few long setae. Legs covered by brown setae, more densely on trochanters, femora and tibiae; femora and tibiae also with rows of longer, thicker, black setae. Fore tibia slightly widened distally, weakly concave near apex. Trochanters without spines. Hind femur with row of 10–11 short spines on proximal third, the last one sometimes slightly longer than the others; distal 2/3 with two parallel rows of spines, dorsalmost row with 11–12 spines, the first and tenth or eleventh larger than the others, ventralmost row with 5 short spines (Figs 1d, 3a). Hind tibia arched, with two parallel rows of about 12–15 subequal short spines, a longer subapical spine and a straight apical spur (Fig. 1d). Dorsum of abdomen densely covered by setae, longer and more numerous on posterior segments. Abdominal sterna with faint longitudinal median carina; II-VI with long golden setae medially; sternum VII with a tuft of setae medially on anterior region (Fig. 1e). Terminalia covered by long setae. Proctiger with rounded apex and lateral projections near middle. Parameres symmetrical, shape as in Fig. 4b.

**Apterous female** (Fig. 2). Similar to apterous male in colour and structure, except for: hind femur much narrower (Table 1), with smaller yellowish marks; proximal half without spines, distal half with a decreasing row of about 7 spines (Fig. 2a, b). Hind tibia straight, without spines throughout length, with apical spur (Fig. 2a, b). Abdominal laterotergites more elevated than in males; last segment with a tuft of setae posteriorly (Fig. 2a). Abdominal sterna without median carina; sternum VII yellowish-brown, with a pair of longitudinal light marks, one on each side of mid-line, without tuft of setae medially on anterior region (Fig. 2b).

**Variation**. Fore tibia, hind femur and hind tibia less robust in some males (Table 1). Concavity near apex of male fore tibia may be incipient. Larger pre-apical spine of male hind tibia may be underdeveloped.

#### Diagnosis

Within the *itatiaiana* group, *Rhagoveliabispoi* sp. n. is more similar to *R.itaiaiana* Drake, 1953, *R.macta* Drake & Carvalho, 1955 and *R.trepida* Bacon, 1948, with which it shares the presence of a medial tuft of setae on the anterior portion of male abdominal sternum VII. However, males of the new species have the main row of spines on the hind femur with two large spines separated by nine or ten smaller spines (Figs 1d, 3a), whereas in *R.itatiaiana* and *R.trepida*, the row consists of a large spine followed by spines gradually decreasing in length towards the apex (as in Fig. 3b). The condition of the row of spines is similar in the new species and *R.macta*; however, they can be distinguished by the mesonotum entirely black in the former (Fig. 1a), but yellowish in the latter (Fig. 5c) and by the shapes of the parameres (compare Fig. 4b, d).

#### Etymology

The new species is named in honour of Dr. Pitágoras da Conceição Bispo, who collected the specimens and also advised CFBF during her doctoral studies.

### Rhagovelia trepida

Bacon, 1948

F538DD43-B582-5A4B-8638-CE446312800A

#### Materials

- **Type status:** Other material. **Occurrence:** individualCount: 13; sex: 5 apterous males, 8 apterous females; occurrenceID: BBB1BEF9-3300-5CBC-9427-DF9ED2759C9E; **Taxon:** order: Hemiptera; family: Veliidae; **Location:** continent: South America; country: Brazil; stateProvince: Paraná; municipality: Balsa Nova / Palmeira; locality: Rio das Pombas, BR-376; decimalLatitude: -25.44; decimalLongitude: -49.79; geodeticDatum: WGS84; **Event:** verbatimEventDate: IV-2017; **Record Level:** type: PhysicalObject; collectionCode: LABIA; basisOfRecord: PreservedSpecimen
- **Type status:** Other material. **Occurrence:** individualCount: 10; sex: 6 apterous males, 4 apterous females; occurrenceID: 37C85CD3-712B-5EC0-B380-3613CE195459; **Taxon:** order: Hemiptera; family: Veliidae; **Location:** continent: South America; country: Brazil; stateProvince: Santa Catarina; municipality: Blumenau; locality: Parque Nacional da Serra de Itajaí, Parque das Nascentes, stream near Lagoa Negra; decimalLatitude: -27.058; decimalLongitude: -49.089; geodeticDatum: WGS84; **Event:** verbatimEventDate: IV-2017; eventRemarks: P.C. Bispo leg.; **Record Level:** type: PhysicalObject; collectionCode: LABIA; basisOfRecord: PreservedSpecimen
- **Type status:** Other material. **Occurrence:** individualCount: 12; sex: 5 apterous males, 7 apterous females; occurrenceID: B21C9802-44CE-5A56-84F6-BAD4235E880A; **Taxon:** order: Hemiptera; family: Veliidae; **Location:** continent: South America; country: Brazil; stateProvince: Santa Catarina; municipality: Rio Negrinho; locality: Rio dos Bugres; decimalLatitude: -26.3722; decimalLongitude: -49.5200; geodeticDatum: WGS84; **Event:** verbatimEventDate: 31.III.2020; eventRemarks: T. Polizei leg.; **Record Level:** type: PhysicalObject; collectionCode: MZUSP; basisOfRecord: PreservedSpecimen

#### Distribution

This species is endemic to the Brazilian Atlantic Forest and is distributed from the coastal areas of the States of Rio de Janeiro and São Paulo to the northern portion of the State of Rio Grande do Sul. It has been seldom collected and reported in only three previous studies (Bacon 1948, Polhemus 1997, Moreira and Barbosa 2011). Above, we present new records of this species from the States of Paraná and Santa Catarina, in southern Brazil (Fig. 6b).

## Identification Keys

### Key to the *Rhagoveliaitatiaiana* group from Brazil

**Table:** 

1	Male abdominal sternum VII with a median tuft of brown setae (as in Fig. 1e)	2
–	Male abdominal sternum VII without median tuft of brown setae	5
2	Mesonotum orange or yellowish at least on central portion (Fig. 5b, c)	3
–	Mesonotum uniformly black (Figs 1a, 2a, 5d)	4
3	Main row of spines on male hind femur with two large spines separated by about eight smaller spines (as in Fig. 3a); male paramere as in Fig. 4d	*Rhagoveliamacta* (Figs 4d, 5c, 6a)
–	Main row of spines on male hind femur with one large spine followed by spines gradually decreasing in length (as in Fig. 3b); male paramere as in Fig. 4c	*Rhagoveliaitatiaiana* (Figs 4c, 5b, 6a)
4	Main row of spines on male hind femur with one large spine followed by spines gradually decreasing in length (Fig. 3b); male paramere as in Fig. 4e	*Rhagoveliatrepida* (Figs 3b, 4e, 5d, 6b)
–	Main row of spines on male hind femur with two large spines separated by nine or ten smaller spines (Figs 1d, 3a); male paramere as in Fig. 4b	*Rhagoveliabispoi* sp. n. (Figs 1, 2, 3a, 4b, 6b)
5	Body length 4.20–4.75 mm; male hind femur without spines on proximal 2/5; abdominal mediotergite I of apterous female elevated with mediotergites II and III; male paramere as in Fig. 4a	*Rhagoveliaaccedens* (Figs 4a, 5a, 6a)
–	Body length 3.30–3.60 mm; male hind femur with an irregular row of spines on proximal 2/5; abdominal mediotergite I of apterous female depressed, mediotergites II–IV elevated; male paramere as in Fig. 4f	*Rhagoveliatrianguloides* (Figs 4f, 5e, 6b)

Modified from Polhemus (1997); apterous males are required or recommended for several steps.

## Supplementary Material

XML Treatment for Rhagovelia bispoi

XML Treatment for Rhagovelia trepida

## Figures and Tables

**Figure 1a.**
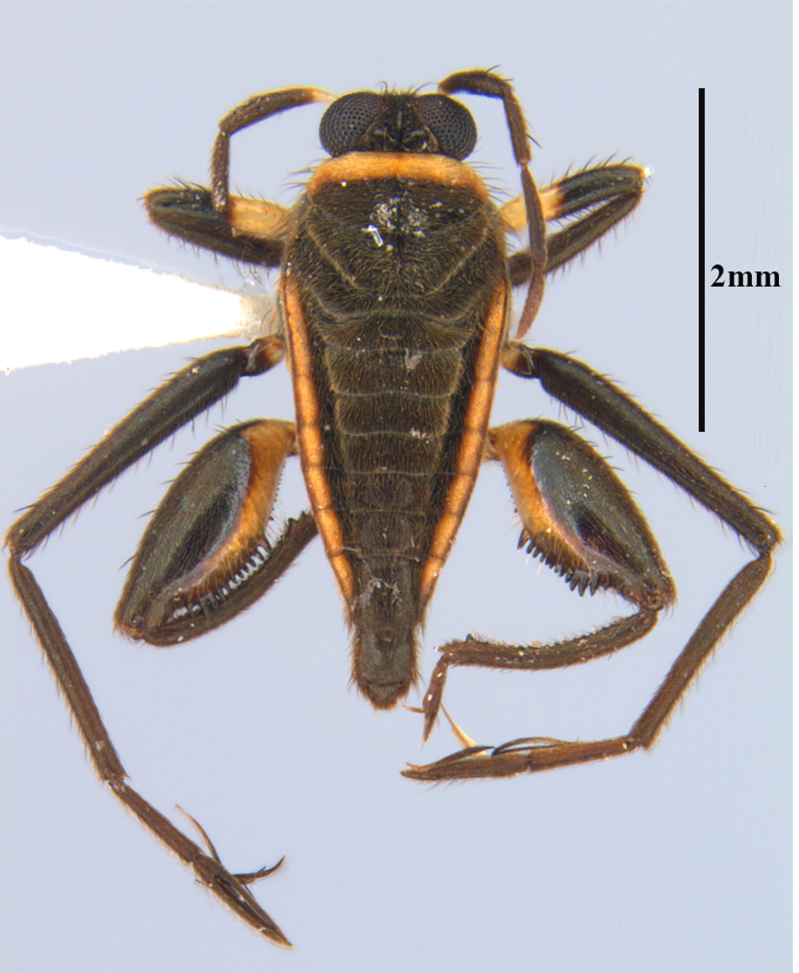
Habitus, dorsal view;

**Figure 1b.**
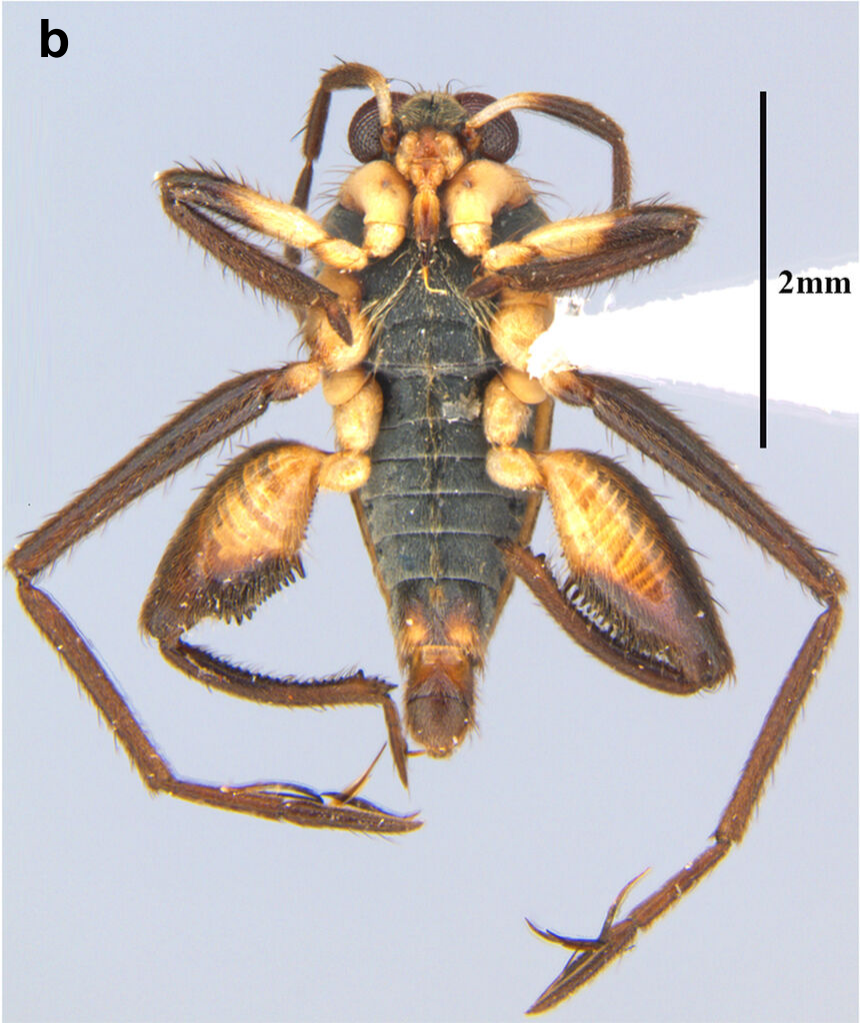
Habitus, ventral view;

**Figure 1c.**
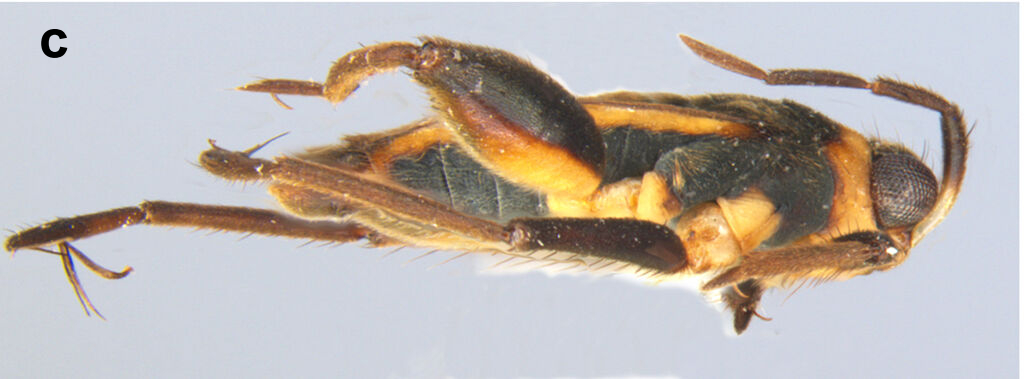
Habitus, lateral view;

**Figure 1d.**
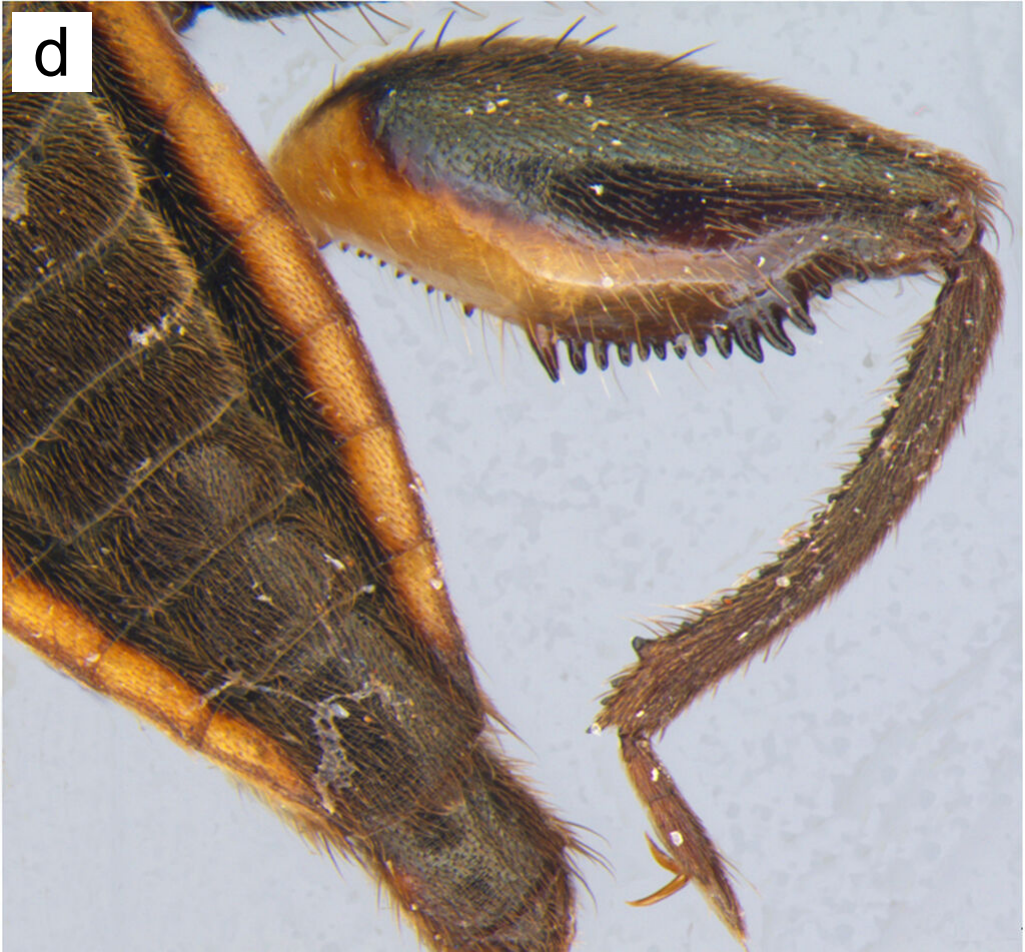
Detail of hind leg, dorsal view;

**Figure 1e.**
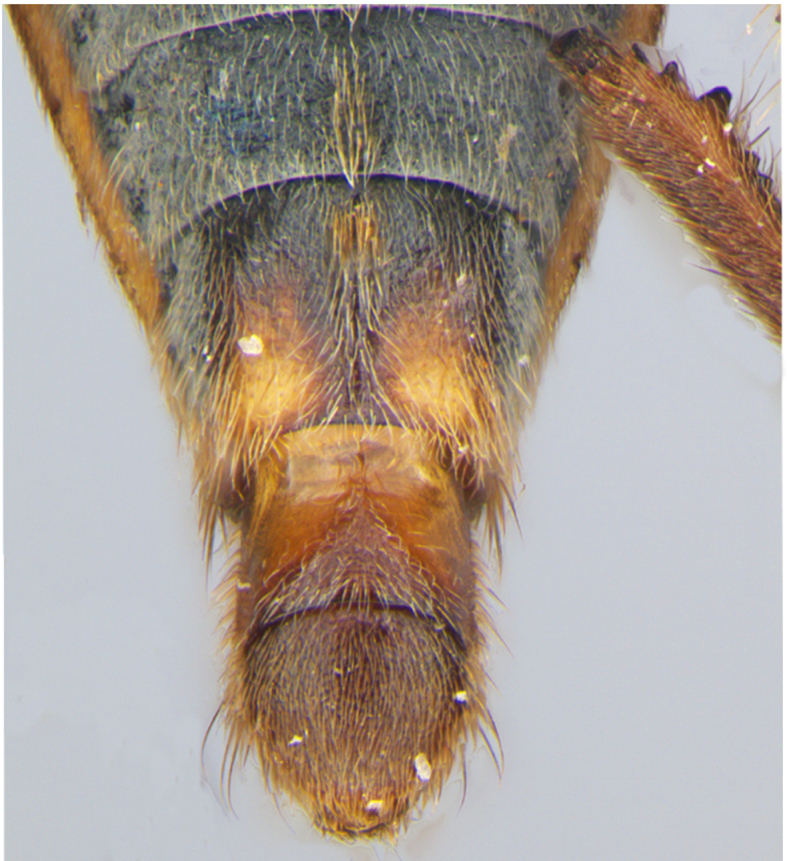
Apex of abdomen, ventral view.

**Figure 2a.**
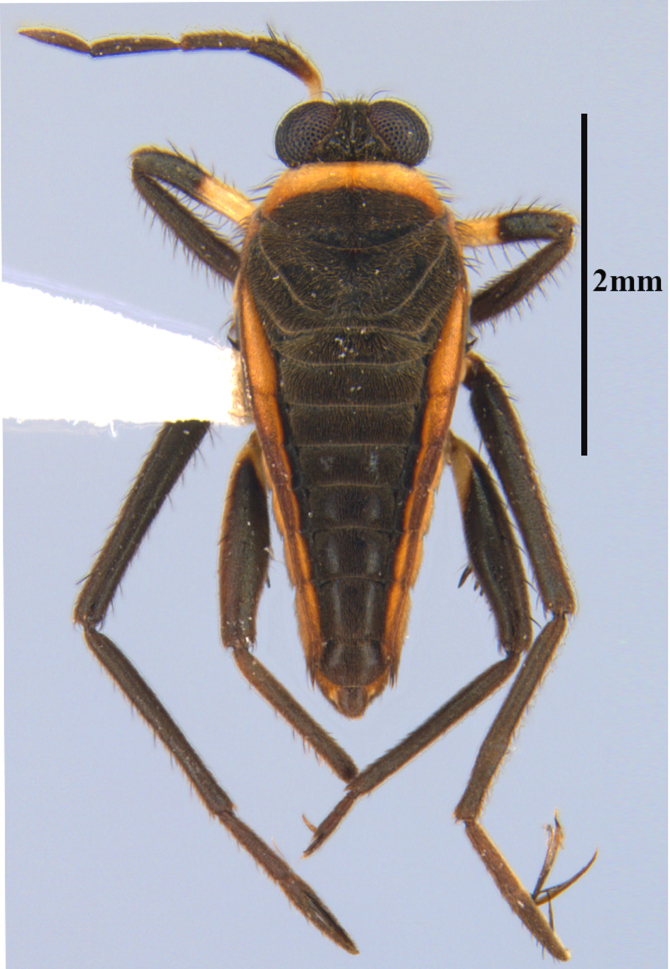
Dorsal view;

**Figure 2b.**
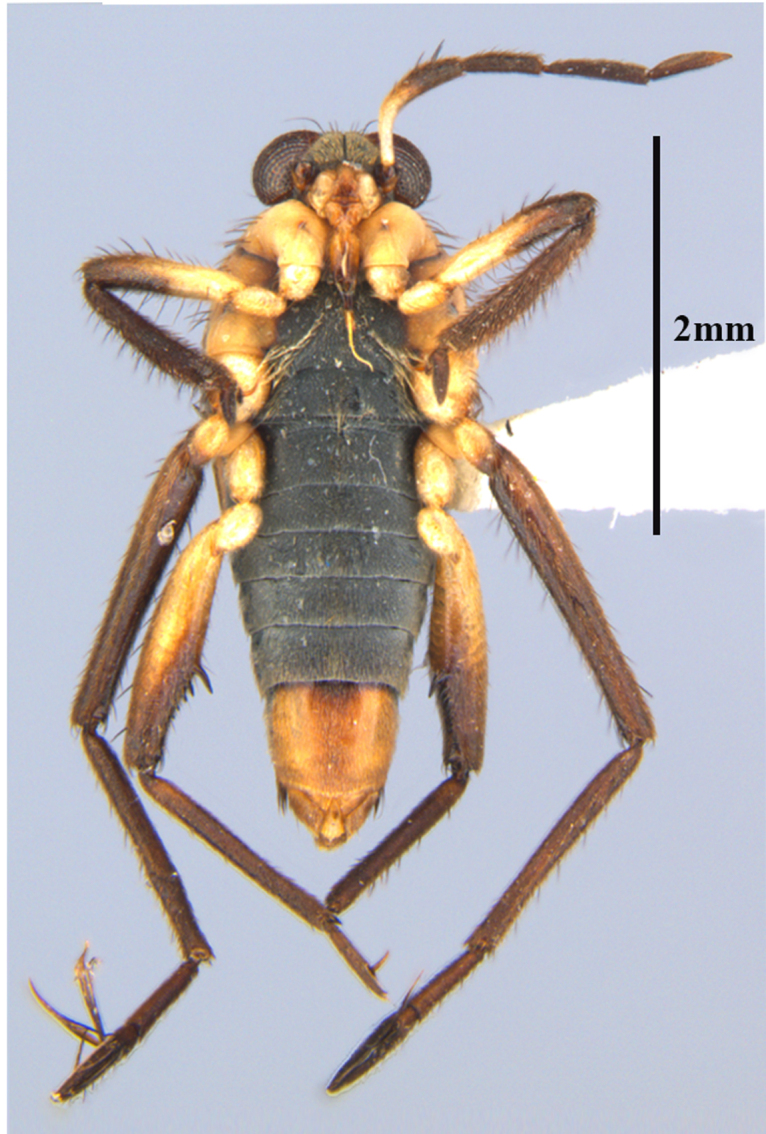
Ventral view;

**Figure 2c.**
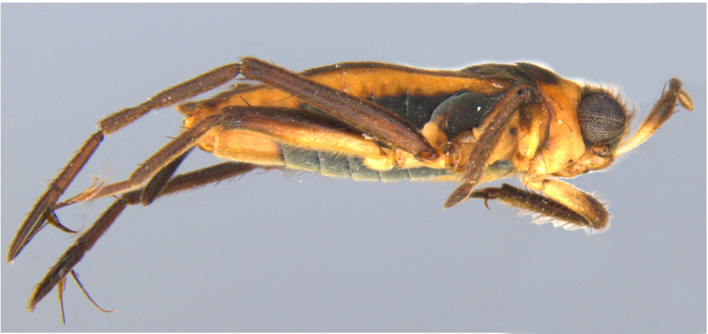
Lateral view.

**Figure 3a.**
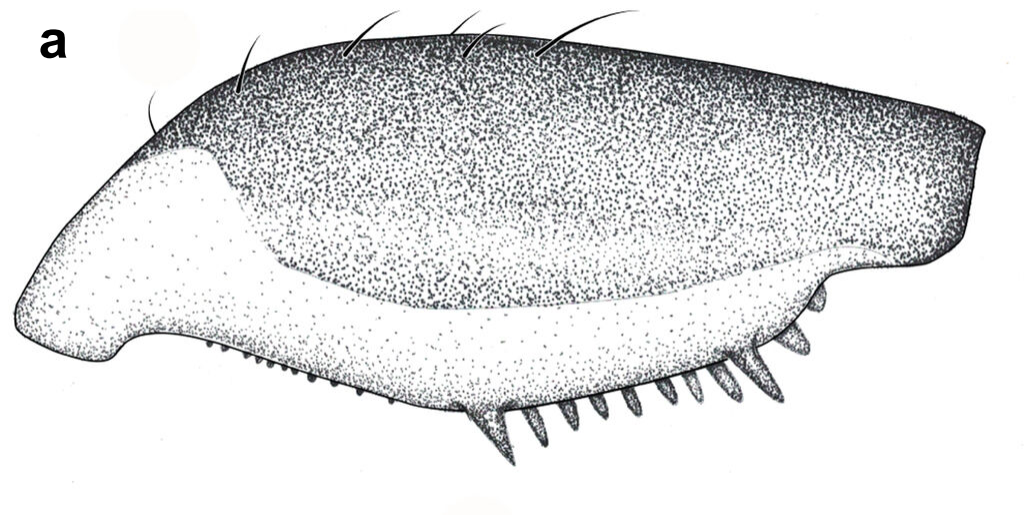
*Rhagoveliabispoi*, sp. n.;

**Figure 3b.**
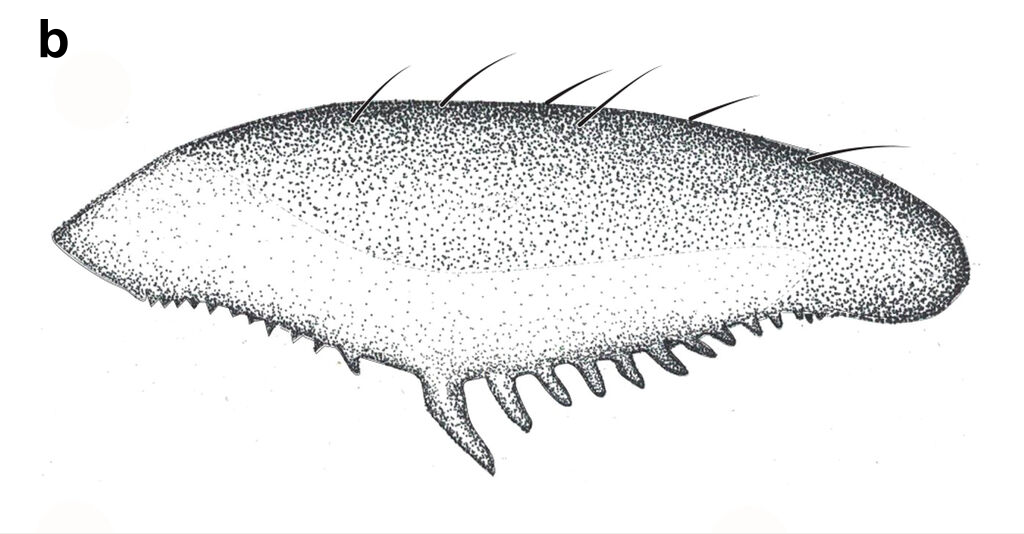
Rhagoveliatrepida.

**Figure 4a.**
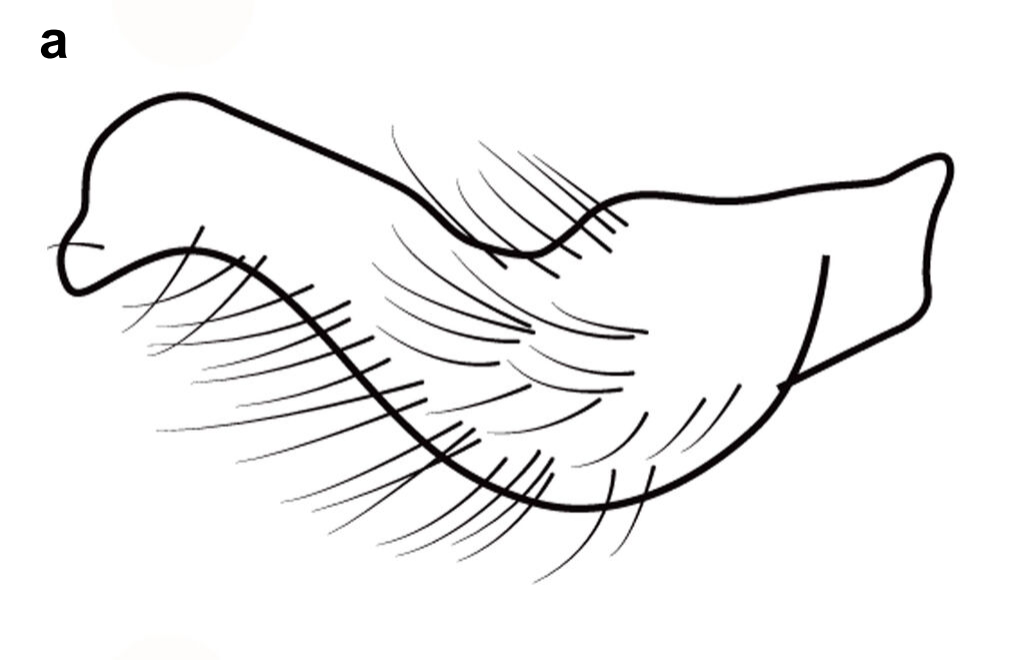
*Rhagoveliaaccedens*, modified from Polhemus (1997);

**Figure 4b.**
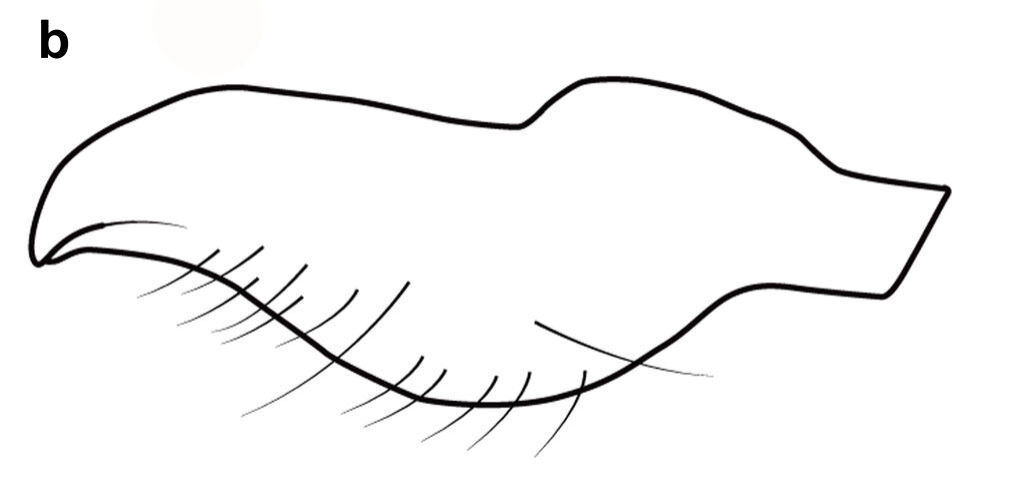
*Rhagoveliabispoi*, sp. n.;

**Figure 4c.**
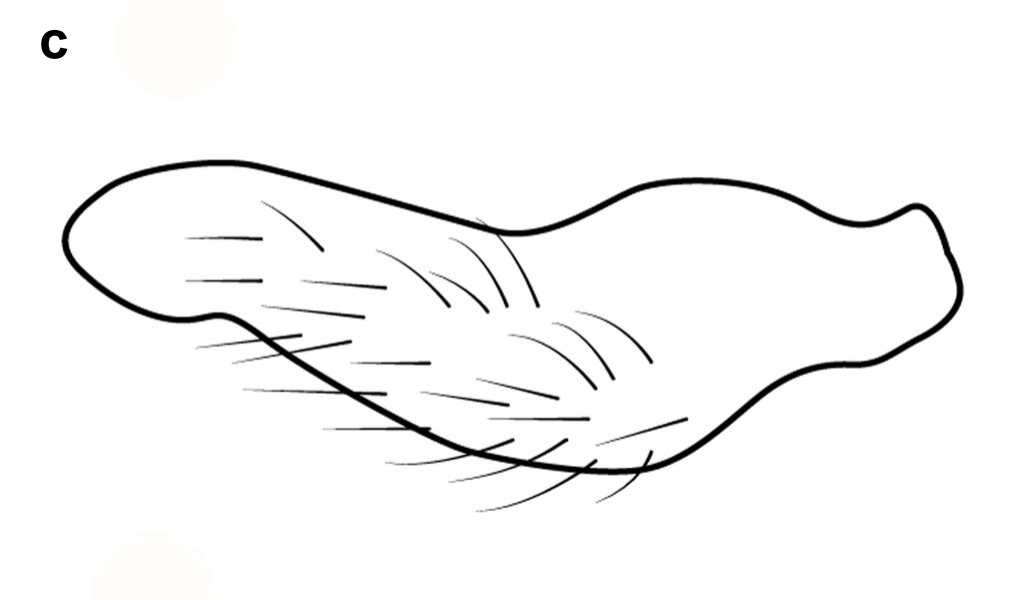
*Rhagoveliaitatiaiana*, modified from Polhemus (1997);

**Figure 4d.**
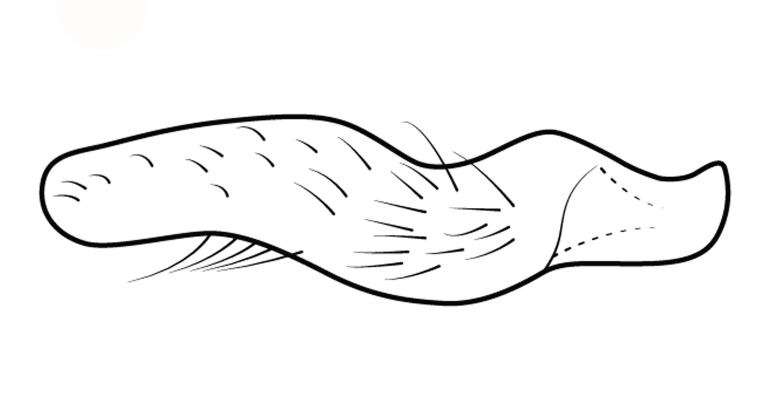
*Rhagoveliamacta*, modified from Polhemus (1997);

**Figure 4e.**
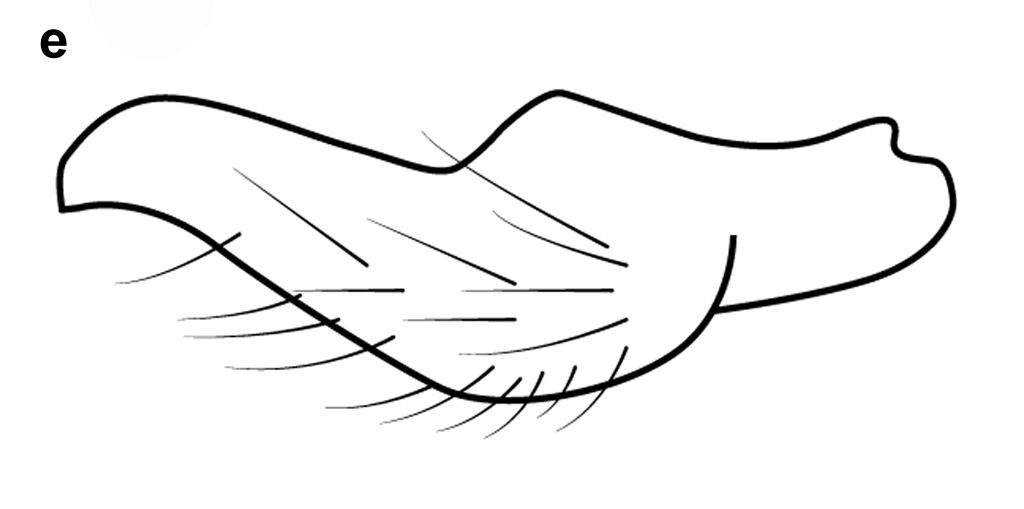
*Rhagoveliatrepida*, modified from Polhemus (1997);

**Figure 4f.**
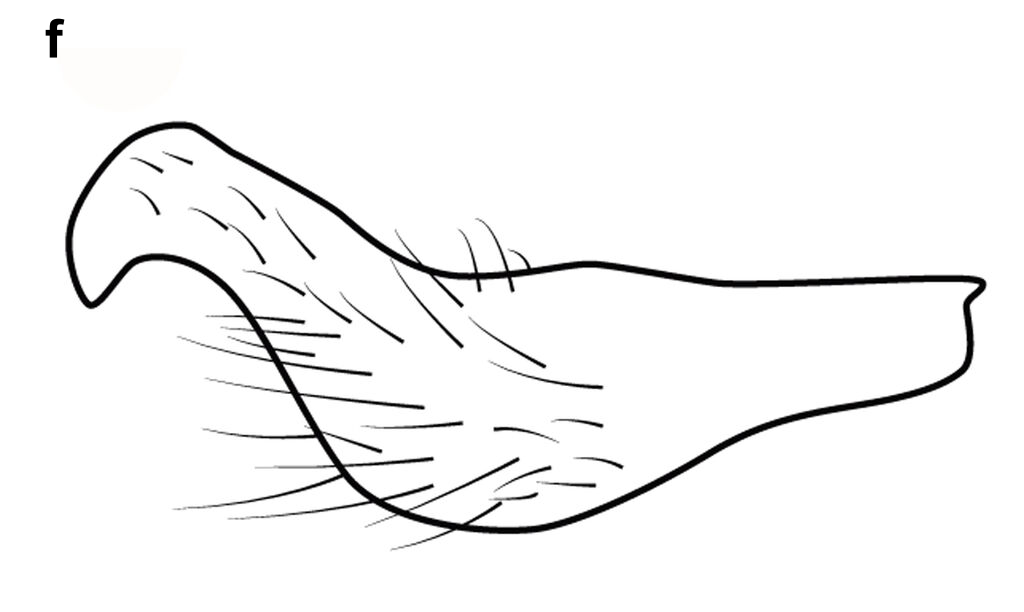
*Rhagoveliatrianguloides*, modified from Nieser and Polhemus (1999).

**Figure 5a.**
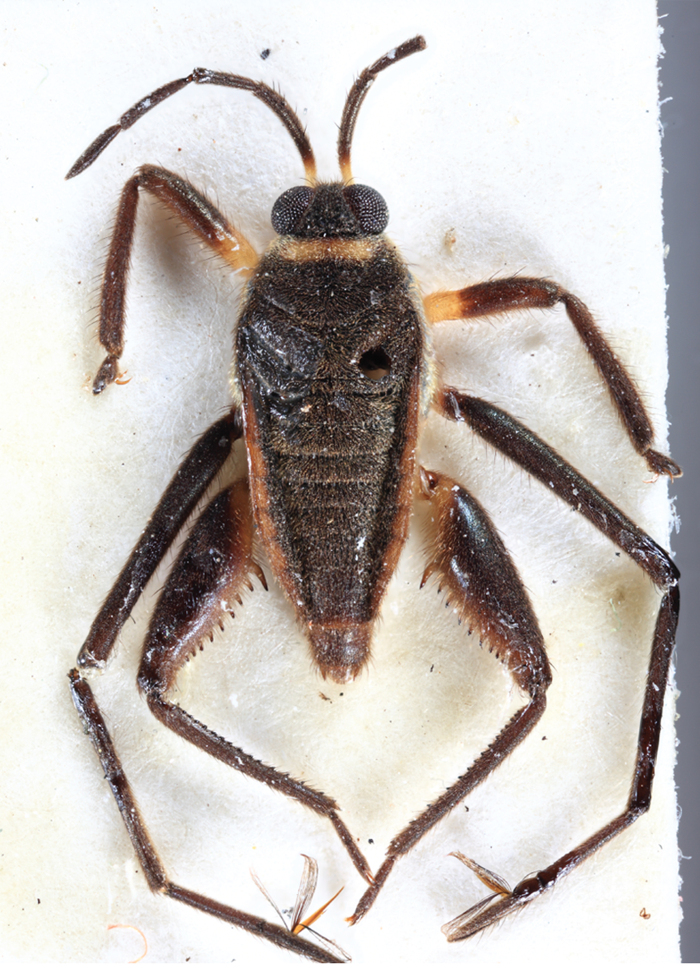
*Rhagoveliaaccedens*, holotype deposited at the NMNH;

**Figure 5b.**
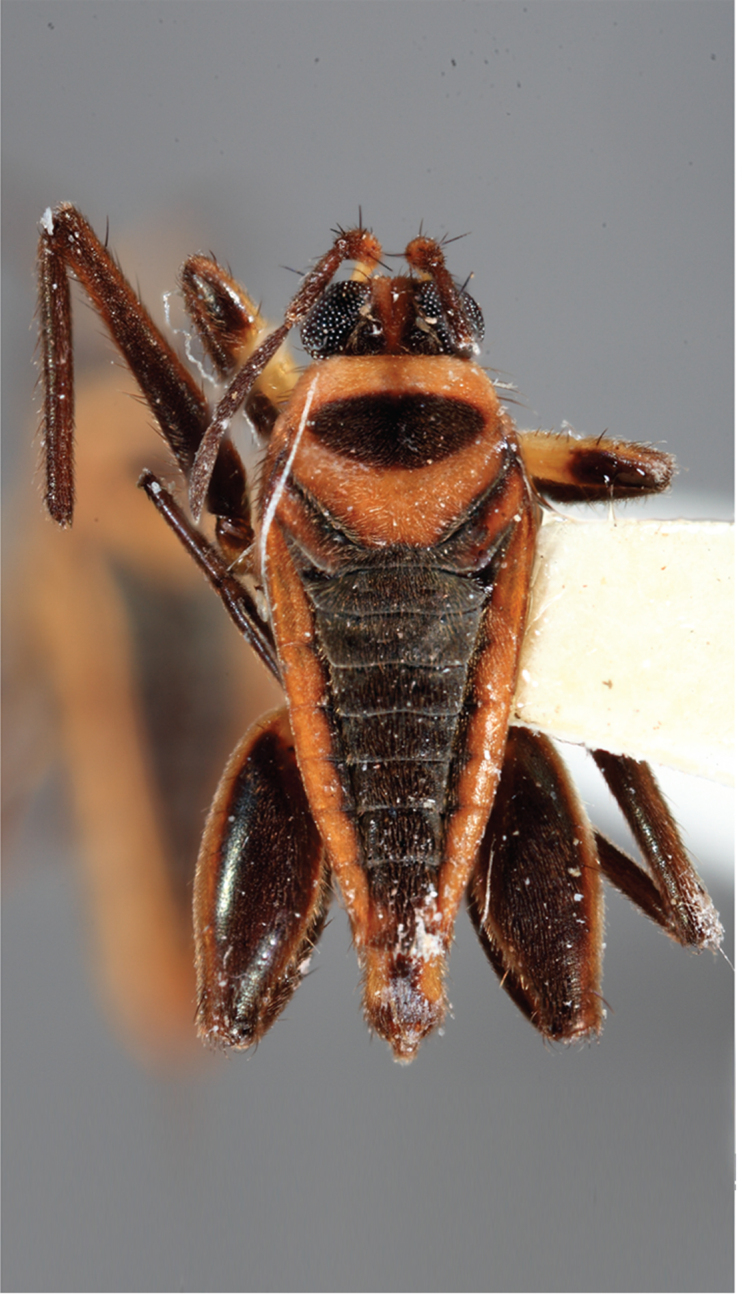
*Rhagoveliaitatiaiana*, holotype deposited at the NMNH;

**Figure 5c.**
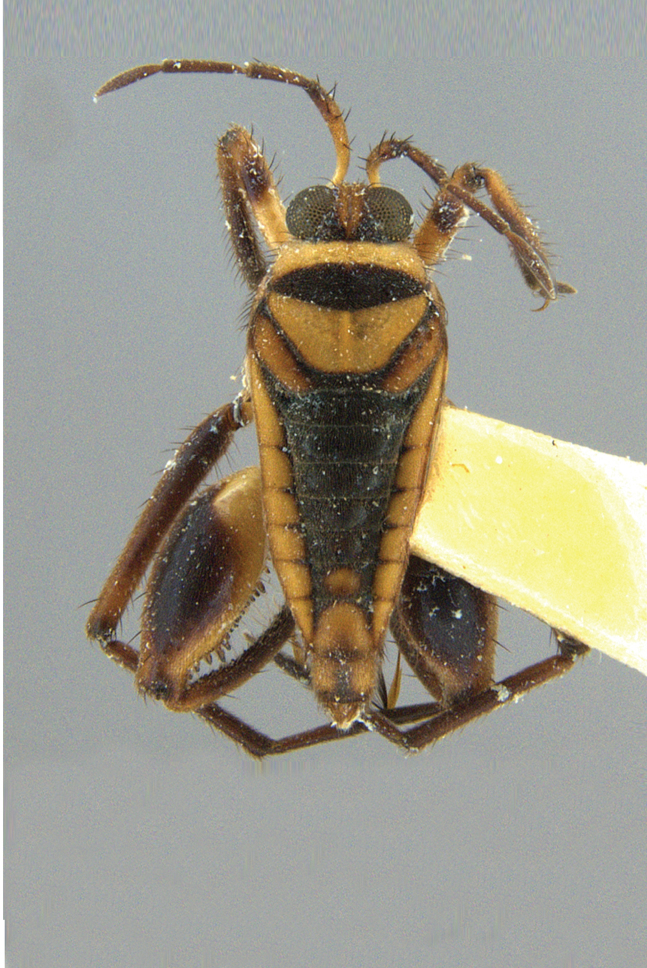
*Rhagoveliamacta*, holotype deposited at the MNRJ, now destroyed;

**Figure 5d.**
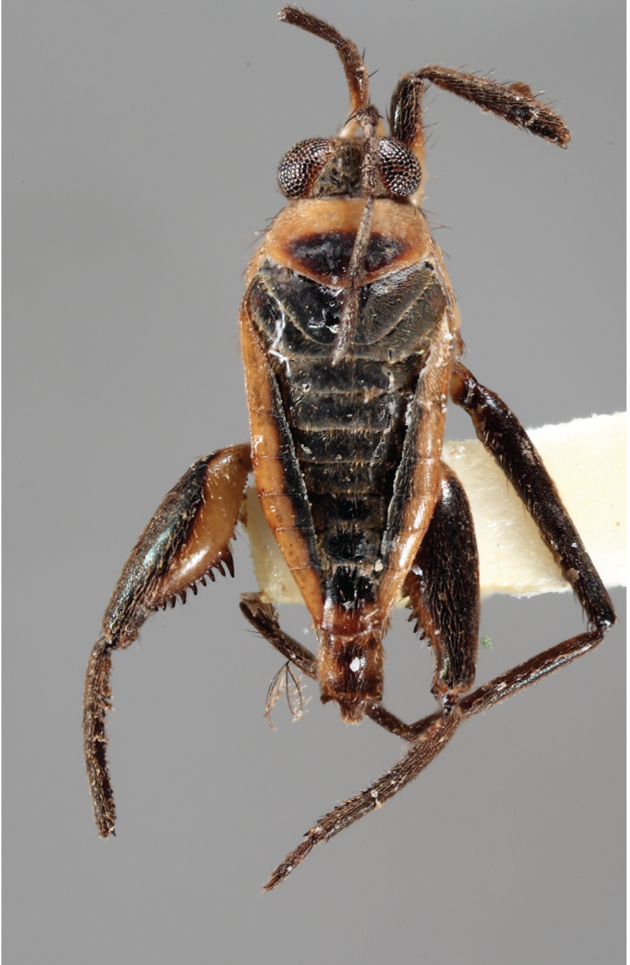
*Rhagoveliatrepida*, paratype deposited at the NMNH;

**Figure 5e.**
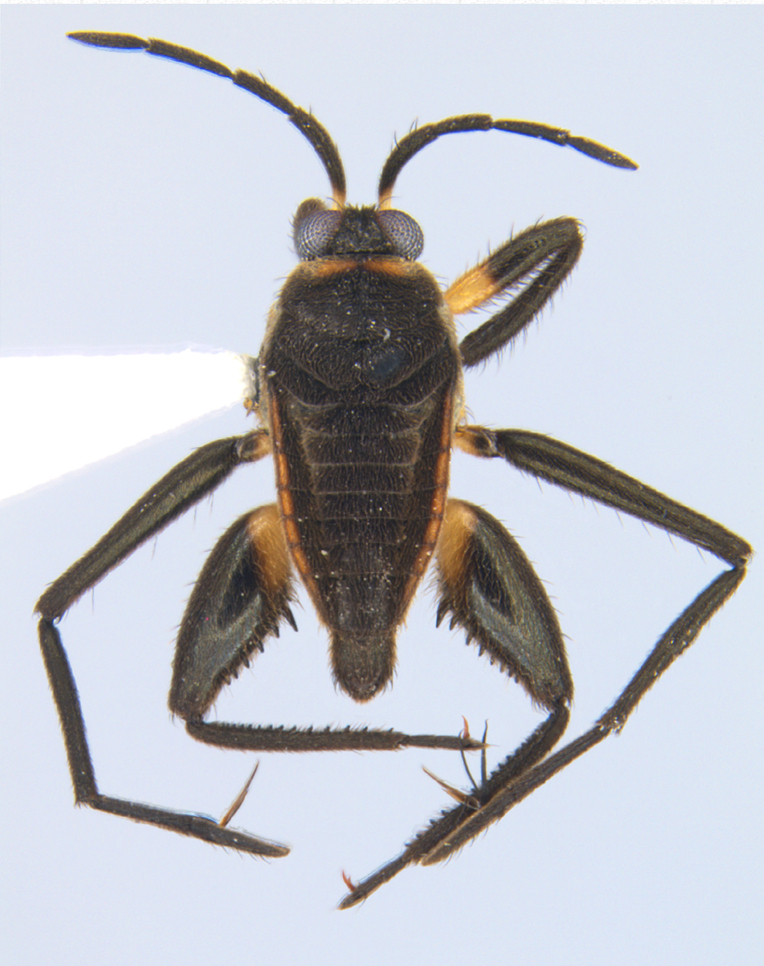
*Rhagoveliatrianguloides*, specimen deposited at the CEIOC.

**Figure 6a.**
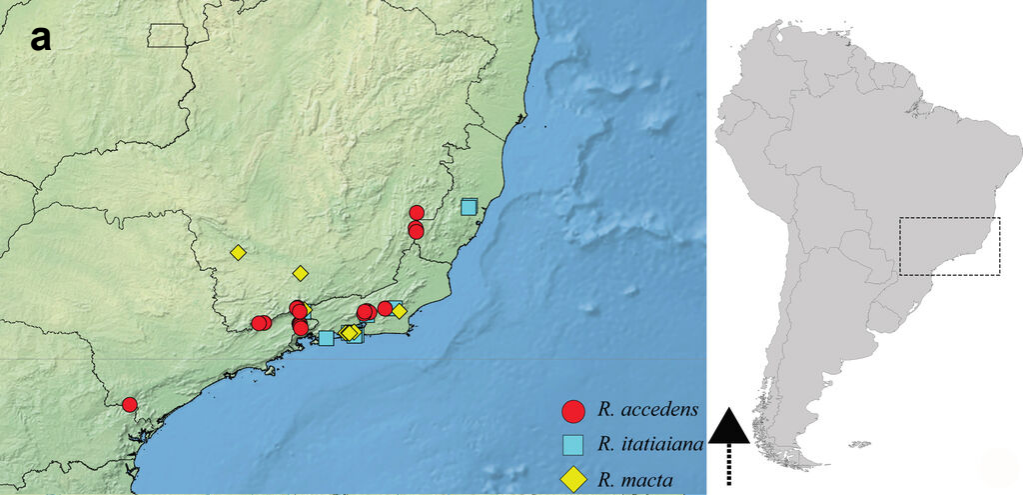
*Rhagoveliaaccedens*, *R.itatiaiana* and *R.macta*;

**Figure 6b.**
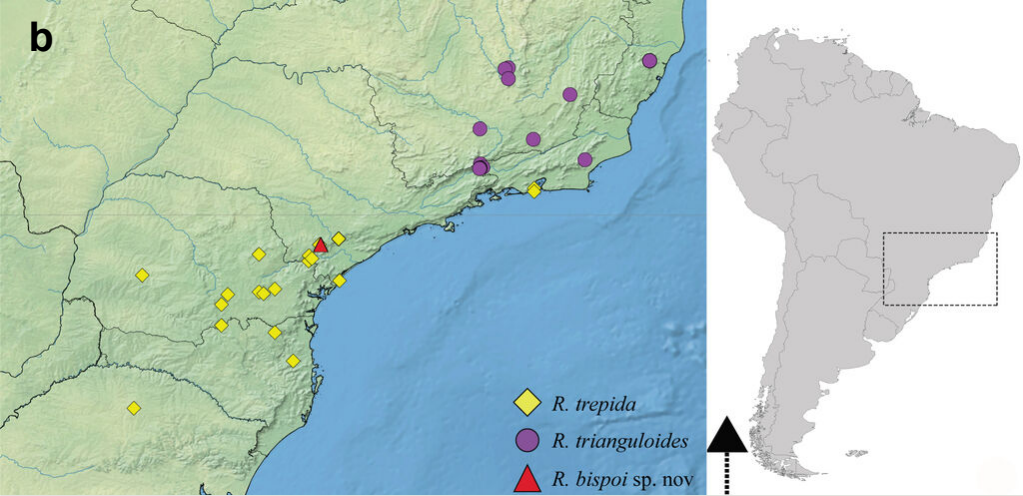
*Rhagoveliatrepida*, *R.trianguloides* and *R.bispoi*, sp. n.

**Table 1.** Measurements obtained from the holotype and five male and five female paratypes of *Rhagoveliabispoi*, sp. n.

		**Paratypes**					
		**Males**			**Females**		
**Structure**	**Male holotype**	**Min**	**Mean**	**Max**	**Min**	**Mean**	**Max**
Body length	3.58	3.58	3.70	3.83	3.63	3.67	3.79
Head length	0.36	0.32	0.35	0.39	0.32	0.32	0.32
Head width through eyes	0.84	0.89	0.96	1.00	0.95	0.95	0.95
Length of antennomere I	0.63	0.63	0.68	0.74	0.58	0.59	0.63
Length of antennomere II	0.42	0.42	0.49	0.56	0.37	0.39	0.42
Length of antennomere III	0.58	0.49	0.53	0.58	0.51	0.52	0.53
Length of antennomere IV	0.47	0.47	0.51	0.53	0.42	0.49	0.53
Maximum eye width	0.37	0.29	0.32	0.39	0.26	0.27	0.29
Pronotum length at mid-line	0.50	0.53	0.52	0.56	0.47	0.51	0.58
Pronotum width	1.05	1.05	1.12	1.21	1.05	1.09	1.16
Length of fore femur	1.16	0.95	1.00	1.06	0.89	0.93	1.00
Length of fore tibia	1.00	1.00	1.04	1.06	0.95	0.98	1.05
Maximum width of fore tibia	0.21	0.13	0.20	0.24	0.11	0.12	0.13
Length of fore tarsomere I	0.05	0.05	0.05	0.05	0.05	0.06	0.07
Length of fore tarsomere II	0.05	0.05	0.05	0.05	0.05	0.06	0.06
Length of fore tarsomere III	0.58	0.52	0.57	0.63	0.58	0.61	0.68
Length of middle femur	1.84	1.74	1.79	1.84	1.53	1.59	1.68
Length of middle tibia	1.47	1.37	1.42	1.48	1.31	1.34	1.37
Length of middle tarsomere I	0.05	0.04	0.05	0.07	0.05	0.05	0.05
Length of middle tarsomere II	0.64	0.64	0.65	0.66	0.52	0.53	0.53
Length of middle tarsomere III	0.82	0.80	0.82	0.83	0.74	0.76	0.84
Length of hind femur	1.63	1.58	1.61	1.67	1.11	1.18	1.32
Maximum width of hind femur	0.74	0.46	0.65	0.74	0.42	0.43	0.45
Length of hind tibia	1.21	1.11	1.21	1.28	1.16	1.21	1.32
Maximum width of hind tibia	0.15	0.07	0.13	0.17	0.07	0.08	0.10
Length of hind tarsomere I	0.06	0.05	0.06	0.07	0.03	0.05	0.06
Length of hind tarsomere II	0.16	0.14	0.15	0.16	0.10	0.11	0.15
Length of hind tarsomere III	0.37	0.33	0.35	0.37	0.31	0.33	0.35

## References

[B9636210] Bacon John A. (1948). Some new species of *Rhagovelia* (Hemiptera, Veliidae). Journal of the Kansas Entomological Society.

[B9636087] Galindo-Malagón Ximena Alejandra, Morales Irina, Moreira Felipe Ferraz Figueiredo (2021). Revision of the *Rhagovelia* angustipes complex (Insecta: Hemiptera: Veliidae) from Colombia. Zootaxa.

[B9636096] Galindo-Malagón Ximena Alejandra, Mondragón-F. Silvia Patricia, Morales Irina, Moreira Felipe Ferraz Figueiredo (2022). New species, synonymies and records in the genus *Rhagovelia* Mayr, 1865 (Hemiptera: Heteroptera: Veliidae) from Colombia. Zootaxa.

[B9861591] Matsuda Ryuichi (1956). A supplementary taxonomic study of the genus *Rhagovelia* (Hemiptera, Veliidae) of the Western Hemisphere. A deductive method. University of Kansas Science Bulletin.

[B9636219] Moreira Felipe Ferraz Figueiredo, Barbosa Juliana Freires (2011). The Veliidae (Hemiptera: Heteroptera: Gerromorpha) from São Paulo State, Brazil: new species, description of the male of *Microveliaioana* Drake & Hottes, 1952, and synonymical and distributional notes. Annales de Limnologie - International Journal of Limnology.

[B9636054] Moreira Felipe Ferraz Figueiredo, Barbosa Julianna Freires, Ribeiro José Ricardo Inacio (2012). Veliidae (Insecta, Heteroptera, Gerromorpha) from southeastern Brazil: three new species from Rio de Janeiro State, a new species group for Neotropical *Rhagovelia* Mayr, and notes on distribution and synonymy. Revista Brasileira de Entomologia.

[B9636033] Moreira Felipe Ferraz Figueiredo, Panizzi A. R., Grazia J. (2015). True bugs (Heteroptera) of the neotropics.

[B9636063] Moreira Felipe Ferraz Figueiredo Veliidae in Catálogo Taxonômico da Fauna do Brasil. PNUD.. http://fauna.jbrj.gov.br/fauna/faunadobrasil/1596.

[B9636308] Nieser Nico, Polhemus Dan A. (1999). Four new species of *Rhagovelia* (Heteroptera: Veliidae) from Minas Gerais (Brazil), with a key to the regional species of the *angustipes* complex. Aquatic Insects.

[B9636071] Padilla-Gil Dora Nancy (2012). Los Hemípteros Acuáticos del Municipio de Tumaco (Nariño, Colombia). Guía Ilustrada.

[B9636016] Padilla-Gil Dora Nancy, Moreira Felipe Ferraz Figueiredo (2013). Checklist, taxonomy and distribution of the *Rhagovelia* Mayr, 1865 (Hemiptera: Heteroptera: Veliidae) of the Americas. Zootaxa.

[B9636079] Padilla-Gil Dora Nancy (2019). Nuevas especies de *Rhagovelia*, *Microvelia*, *Buenoa*. Registros Nuevos de Otros Heterópteros de Colombia (Gerromorpha, Nepomorpha, Leptopodomorpha).

[B9636046] Polhemus Dan A. (1997). Systematics of the genus *Rhagovelia* Mayr (Heteroptera: Veliidae) in the Western Hemisphere (exclusive of the *angustipes* complex).

[B9636007] Polhemus John T., Polhemus Dan A. (2008). Global diversity of true bugs (Heteroptera; Insecta) in freshwater. Hydrobiologia.

